# Supplementary material for: A novel alpha-synuclein K58N missense variant in a patient with Parkinson’s disease
Source: medRxiv. 2025 Feb 14:2025.02.07.25321793. Preprint. [Version 2] doi: 10.1101/2025.02.07.25321793 (PMC11844588; doi:10.1101/2025.02.07.25321793)
Supplement: Supplement 1 [file media-1.pdf]

A novel alpha-synuclein K58N missense variant in a patient with Parkinson's disease

Mohammed Al-Azzani[1†], Sandrina Weber[2†], Nagendran Ramalingam[3], Maria Ramón[1], Liana Shvachiy[1], Gonçalo Mestre[1], Michael Zech[4,5,6], Kevin Sicking[7,8], Alain Ibáñez de Opakua[9], Vidyashree Jayanthi[3], Leslie Amaral[1,10], Aishwarya Agarwal[11], Aswathy Chandran[11], Susana R. Chaves[10], Juliane Winkelmann[4,5,12,13], Claudia Trenkwalder[14,15], Maike Schwager[16], Silke Pauli[16], Ulf Dettmer[3], Claudio O. Fernández[17], Janin Lautenschläger[11], Markus Zweckstetter[9,18], Ruben Fernandez Busnadiego[7,8,19,20], Brit Mollenhauer[2,15*], and Tiago Fleming Outeiro[1,9,21,22*]

Supplementary Materials

This PDF file includes:

Figs. S1 to S6

Fig. S1.

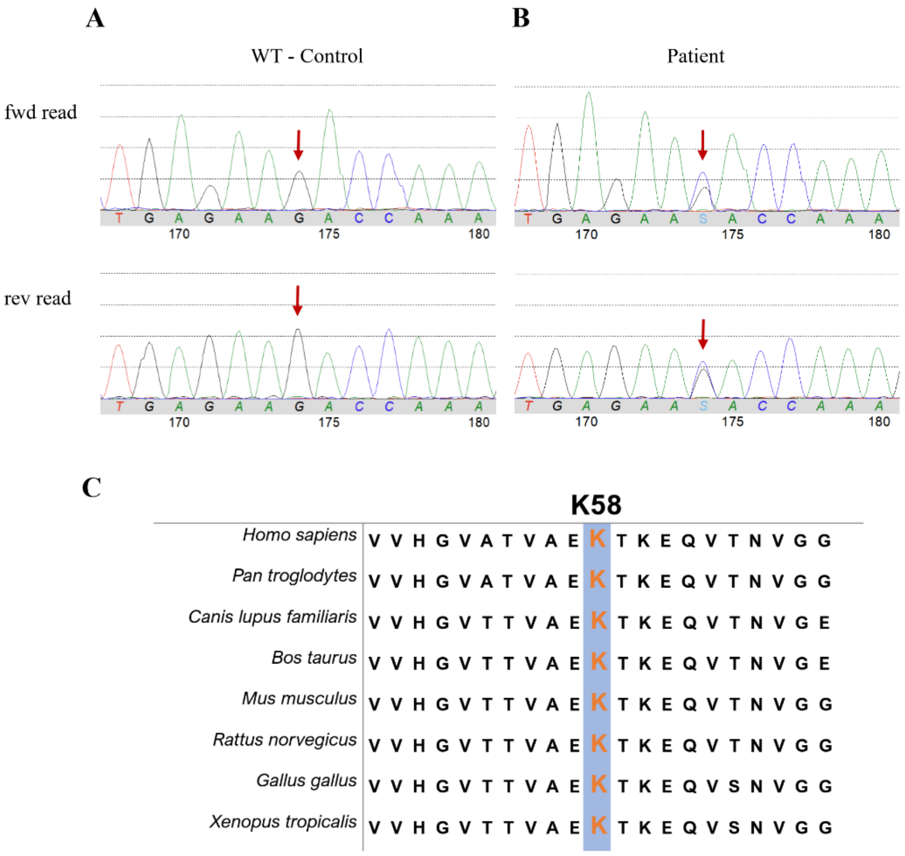

Fig. S1. Identification of K58N mutation. (A) and (B) present DNA sequencing results of the SNCA gene, Exon 4, from a wildtype control sample (A) and a patient sample (B) with a heterozygous variant c.174G>C; S = G or C. (C) illustrates conservation of the SNCA K58N missense mutation across various species.

Fig. S2.

A

Parameters	WT aSyn	K58N aSyn
Length	140	140
#Amyloids	20	20
Best energy	-7.239327	-7.239327
% disorder	34.28	34.28
% α-helix	26.43	20.71
% β-strand	22.14	25.71
%coil	51.43	53.57

B

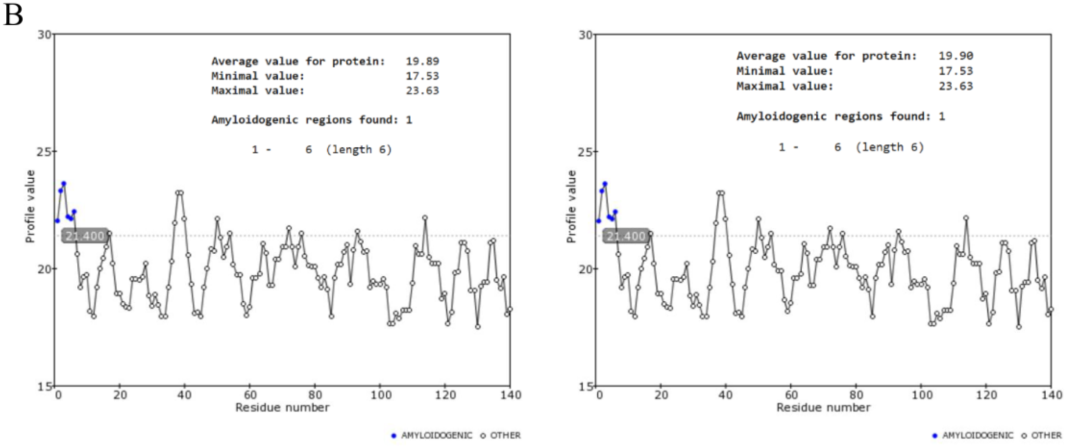

C

No.	Peptide	Amyloid probability	Non-amyloid probability
53	ATVAEK	0.740	0.260
54	TVAEKT	0.172	0.828
55	VAEKTK	0.971	0.029
56	AEKTKE	0.823	0.177
57	EKTKEQ	0.989	0.011
58	KTKEQV	0.999	0.001

D

No.	Peptide	Amyloid probability	Non-amyloid probability
53	ATVAEN	0.992	0.008
54	TVAENT	0.771	0.229
55	VAENTK	0.954	0.046
56	AENTKE	0.024	0.976
57	ENTKEQ	0.999	0.001
58	NTKEQV	1.000	0.000

Fig. S2. Prediction of the impact of the K58N mutation on structural and aggregation properties of aSyn. (A) evaluation of the effect of K58N using PASTA 2.0 algorithm. The mutation tends to reduce the amount of α-helix and number coils, and to increase the β-strand content. (B) Prediction of amyloidogenic sequences of WT (left) and K58N (right) aSyn through FoldAmyloid tool based on the probability of formation of hydrogen bonds. A slight increase in the aggregation probability was observed for K58N variant. (C) Analysis of aggregation-prone

regions for WT and and K58N aSyn (D) utilizing GAP (Aggregation Proneness) prediction algorithm, which is based on analyzing each part of the protein as a 6-amino acid long peptide and indicating which is the probability of the peptide forming an amyloid structure. According to GAP, there is an increase in the individual probability of each peptide to form amyloid structures, with exception to the peptide in the position 56, for K58N compared to WT aSyn.

Fig. S3.

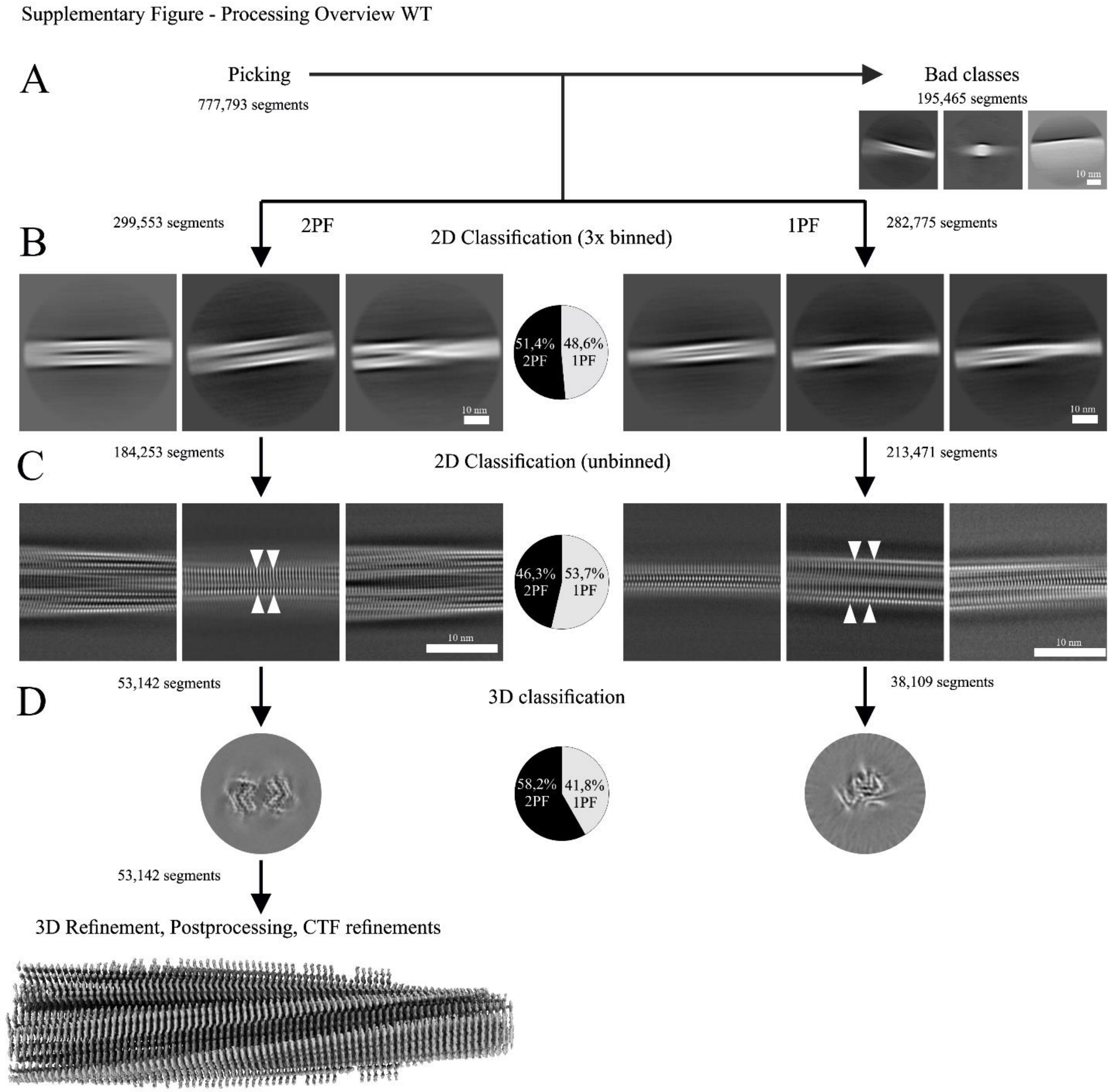

Fig. S3. Detailed Processing Workflow for the WT aSyn Dataset. (A) From the initially picked helical segments, a subset was excluded due to artifacts, such as the presence of carbon edges. The remaining segments were sorted into two categories based on structural features: segments exhibiting two protofilaments (2PF, "wide") and those with a single protofilament (1PF, "narrow"). (B) During the initial classification step, which utilized thrice-binned segments, the distribution of

segments between the two groups was approximately equal, as depicted in the associated pie chart. (C) Further classification performed on unbinned data confirmed the results of the initial classification, with segments evenly divided between the 2PF and 1PF categories, as illustrated in the corresponding pie chart. (D) The three-dimensional classification results are shown, including the final electron density map of the 2PF data following 3D refinement, postprocessing, and CTF refinement. The accompanying pie chart reveals a slightly greater contribution of segments to the 2PF (two-protofilament) structure compared to the 1PF structure.

Fig. S4.

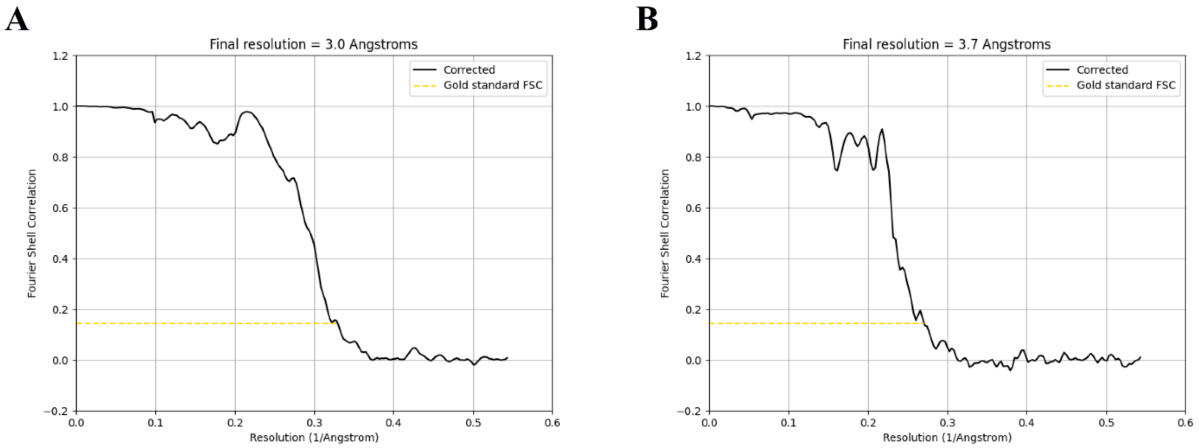

Fig. S4. Fourier Shell Correlation (FSC) curves for 2PF WT (A) and 2PF K58N (B).

Fig. S5.

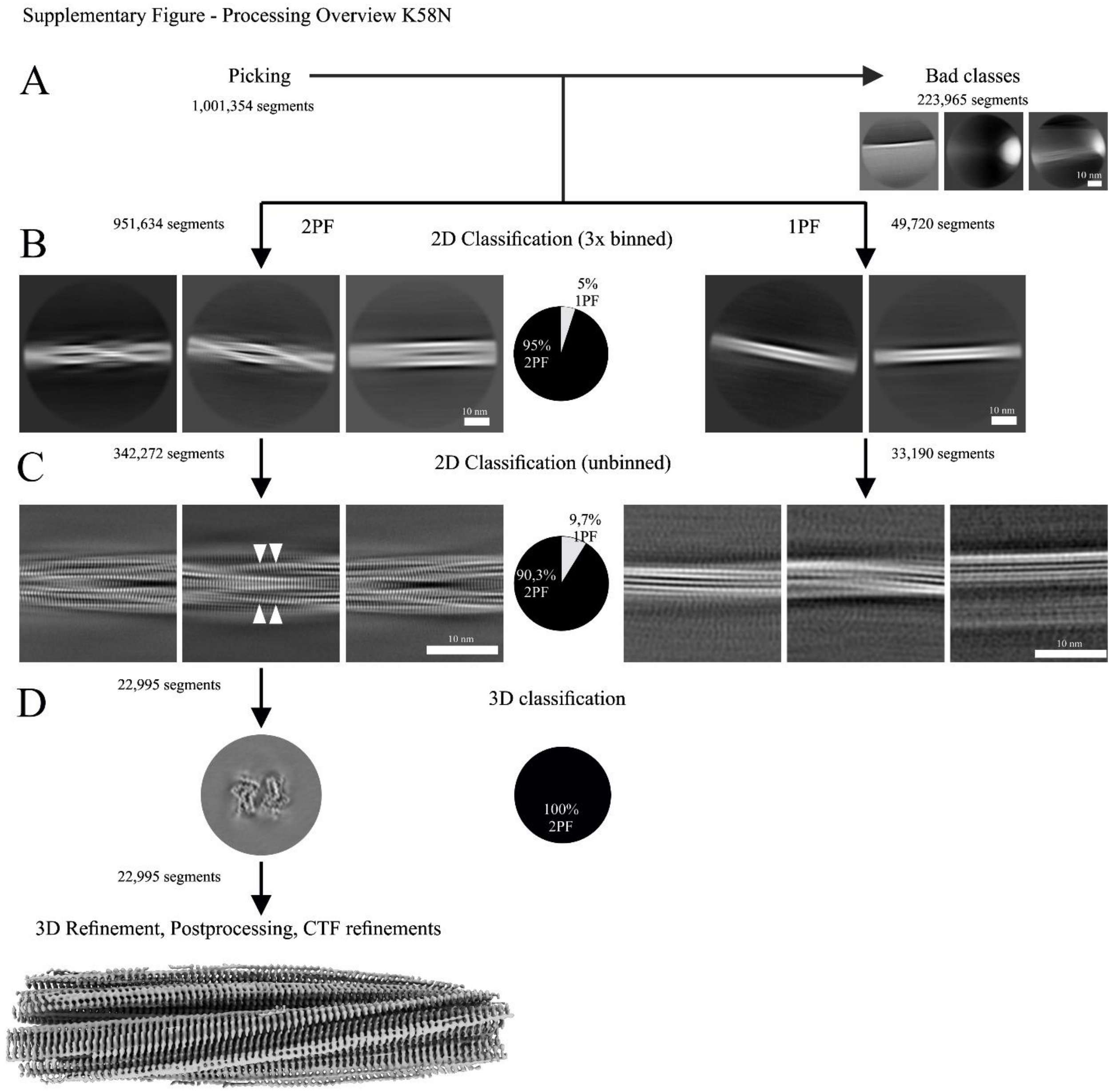

Fig. S5. Detailed Processing Workflow for the K58N aSyn Dataset. (A) A subset of the initially picked helical segments was removed due to artifacts, such as those containing carbon edges. The remaining segments were divided into two categories based on their structural features: segments showing two protofilaments (2PF, "wide") and those showing a single protofilament (1PF, "narrow"). (B) During the initial classification step with three times binned segments, a significant majority of the segments were categorized as 2PF, with only a small fraction classified as 1PF, as illustrated by the provided pie chart. (C) Subsequent classification with unbinned data supported the initial results, as depicted by the adjacent pie chart. In contrast ot the 2PF dataset, no clear beta-sheet separation was observed in the unbinned class averages for the 1PF dataset, hinting at the lower quality and quantity of the 1PF segments. (D) The results of the three-dimensional

classification are presented. For the 2PF data, the refined electron density map is shown after 3D refinement, postprocessing, and CTF refinement. Due to the poor quality of particles and class averages in the 1PF dataset, further processing via 3D classification was not feasible.

Fig. S6.

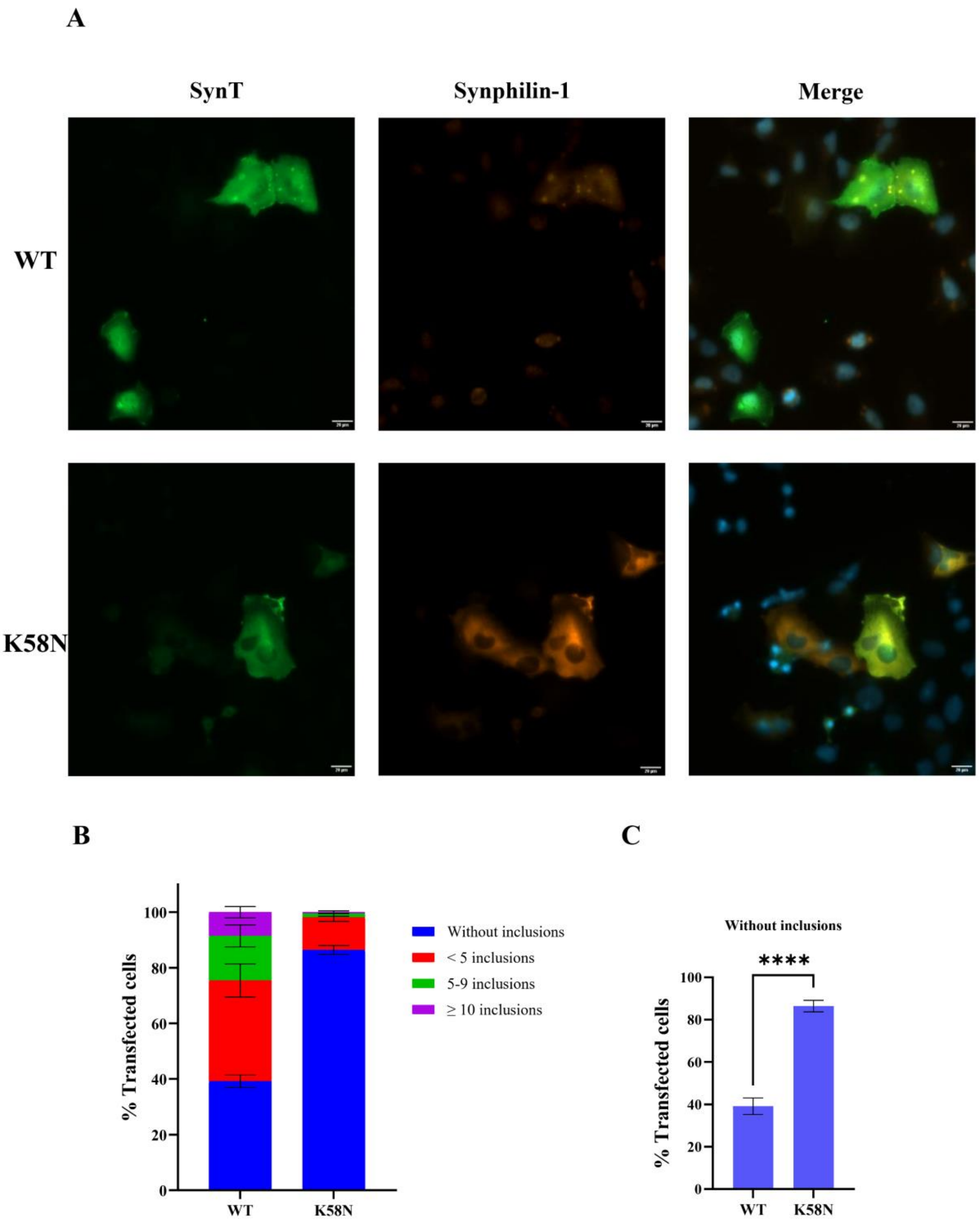

Fig. S6. Effect of K58N mutation on inclusion formation in cells. (A) Representative immunohistochemistry images of H4 cells showing the patterns of inclusion formation for both WT and K58N SynT variants. Scale bar: 20 μm. (B) and (C) show the quantification of the number

of inclusions from transfected cells. A total of 50 cells were counted using a 20x objective for each experiment, and classified into four groups based on their inclusion patterns. Student’s t-test was used to analyze the data, and the results are shown as mean ± SD from 3 independent experiments.
